# Skin Damages—Structure Activity Relationship of Benzimidazole Derivatives Bearing a 5-Membered Ring System

**DOI:** 10.3390/molecules25184324

**Published:** 2020-09-21

**Authors:** Ernestine Nicaise Djuidje, Elisa Durini, Sabrina Sciabica, Elena Serra, Jan Balzarini, Sandra Liekens, Stefano Manfredini, Silvia Vertuani, Anna Baldisserotto

**Affiliations:** 1 Department of Life Sciences and Biotechnology, Master Course in Cosmetic Science and Technologies, University of Ferrara, 44121 Ferrara, Italy; djdrst@unife.it (E.N.D.); elisa.durini@unife.it (E.D.); mv9@unife.it (S.M.); vrs@unife.it (S.V.); 2 Department of Chemical and Pharmaceutical Sciences, University of Ferrara, 44121 Ferrara, Italy; scbsrn@unife.it; 3 Aptuit, An Evotec Company, 37135 Verona, Italy; Elena.serra@aptuit.com; 4 Department of Microbiology and Immunology, KU Leuven, University of Leuven, Rega Institute for Medical Research, Laboratory of Virology and Chemotherapy, B-3000 Leuven, Belgium; jan.balzarini@kuleuven.be (J.B.); sandra.liekens@rega.kuleuven.be (S.L.)

**Keywords:** benzimidazole, UV-filter, melanoma

## Abstract

In the search for scaffolds for multifunctional compounds we investigated the structure activity relationship of a class of benzimidazole derivatives bearing 5-membered ring. The newly synthesized and the already known compounds were divided into three classes that present different substituent at 5 position of the benzimidazole ring (-H, -COOH or –SO_3_H) and different heterocycle at position 2 (thiophene, furan or pyrrole). All the derivatives were synthesized and tested to determine their photoprotective profile against UV rays, in vitro antioxidant capacity against different radicals (DPPH and FRAP test), antifungal inhibitory activity (dermatophytes and *Candida albicans*), antiviral and antiproliferative activity. A Structure-Activity Relationship study indicated compound **10**, bearing a pyrrole heterocycle on the benzimidazole ring, as the best multifunctional derivative of the series and as potential candidate for the development of drugs especially in case of melanoma.

## 1. Introduction

Skin is the largest body organ constituting an environmental interface by forming a protective barrier essential for homeostasis. For this reason skin is constantly exposed to exogenous and endogenous agents that could altered its functions. UV rays, microbes and pollution are responsible for external damage to the skin. However, UV is the most important factor responsible for the production of reactive oxygen species (ROS) which consequently induces the development of skin cancer and aging. So the discovery of sunscreens with combined photoprotective, antioxidant, antifungal and anti-melanoma activity remains the challenge of researchers in the pharmaceutical and cosmetic fields [1,2,3].

Heterocyclic compounds are fundamental building blocks of various essential natural and synthetic compounds. They play an important role in drug design thanks to their ability to act as both biomimetic and reactive pharmacophores [4]. Many of them are five- and / or six-membered heterocyclic compounds having one to three heteroatoms (especially oxygen, nitrogen and sulfur) in their structure and are significant in organic chemistry, medicinal chemistry and biochemistry [5]. They are often found in large percentage in biomolecules like enzyme, vitamins and natural products [6]; for these reasons heterocyclic compounds remain the challenging issue for the researchers for the development of novel drugs [7]. Moreover, five membered-rings fused with benzene moiety attracted the attention of medicinal chemists especially benzimidazoles due to their diverse biological activities including antitumor [8,9,10,11,12], antifungal [13], anthelmintics [14], anti-inflammatory [15], analgesics [16] and antiviral [17]. Compounds belonging to this class of heterocycles are considered privileged due to their broad spectrum pharmacological activities [18].

In medicinal chemistry, interest in the benzimidazole ring increased in the 1950s after the discovery of 5,6-di-methyl-1-(α-D-ribofuranosyl) benzimidazole as an integral part of vitamin B12. Besides, benzimidazole derivatives have a wide range of biological activities such as antibacterial, antimicrobial, antioxidant, anti-tuberculosis, antimalarial, antiulcer, antiallergic, antidiabetic as well as use in cardiovascular disease [19,20,21,22]. In addition to their therapeutic applications, benzimidazoles are known for their UV filtering ability [23]. Besides, 2-phenyl-5-(pyrrolidin-1-yl)-1-(3,4,5-trimethoxybenzyl)-1H-benzimidazole (PPTMB), 2-[4-(2-{4-[1-(2-Ethoxyethyl)-1H-benzimidazol-2-yl]-1-piperidinyl}ethyl)phenyl]-2-methylpropanoic acid (Bilastine) and 1-[(4-chlorophenyl)methyl]-2-methylbenzimidazole (Chlormidazole) are benzimidazole analogues, respectively used in the treatment of cancer, allergic rhino-conjunctivitis and fungal infection [24].

5-Membered rings including thiophene, furan and pyrrole have received considerable interests in the fields of medicinal chemistry [25]. Some studies showed that chalcone and pyrimidine analogues containing pyrrole, furan and thiophene have increased the antimalarial activity of several prepared drugs [26]. They also have significant inflammatory, antineoplastic and antimicrobial activity [27].

**Figure 1:**
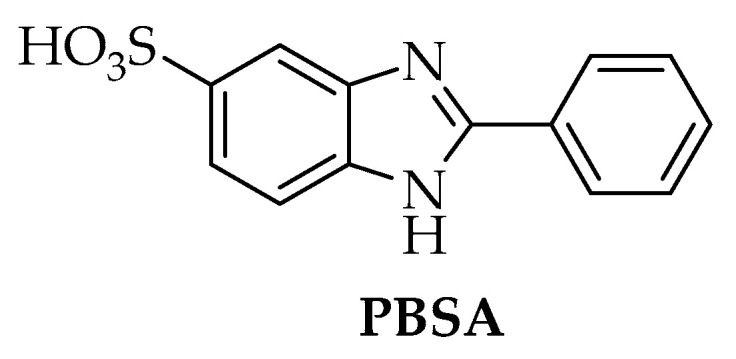
Lead compound.

Based on the biological activities of heterocyclic compounds mentioned above we have directed this work towards the study of the structure activity relationship of benzimidazole bearing 5-membered ring derivatives and their biological study as smart multifunctional compounds for skin damages. To this aim, our interest was in developing compounds starting from 2-phenyl-1H-benzimidazole-5-sulphonic acid (PBSA, a commercial UVB filter, Figure 1) as lead compound. Since the isosteric modification of a lead compound is a key tool in the pharmaceutical sector for the rational design of new drugs, changes have been made to the PBSA to implement this project, in particular to positions 2 by introducing a 5-member ring on the phenyl ring and replacing the functional group (-SO_3_H) in position 5 of the benzimidazole ring with -COOH and –H. The three series explored include both already known and newly synthesized compounds.

## 2. Results and Discussion

### 2.1. Chemistry

Different changes have been made to PBSA in order to improve its UV filter profile and also give it other activities with the aim to obtaining multifunctional compounds. For this purpose, the phenyl ring has been replaced by 5-membered rings (pyrrole, thiophene and furan) which are benzene isosters. The sulfonic acid portion at position 5 of the benzimidazole ring was also modified by carboxylic acid and hydrogen.

**Scheme 1:**
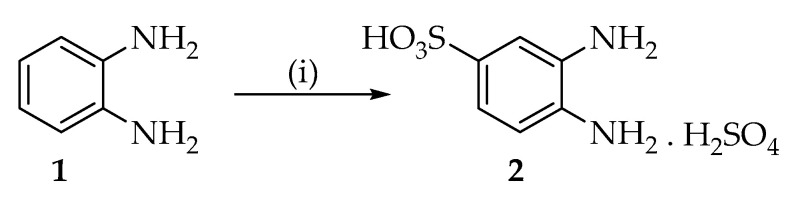
Synthesis of 3,4-diamino-benzene sulfonic acid, sulfate salt. Reagents and conditions: (i) H_2_SO_4_, reflux, 24 h.

**Scheme 2:**
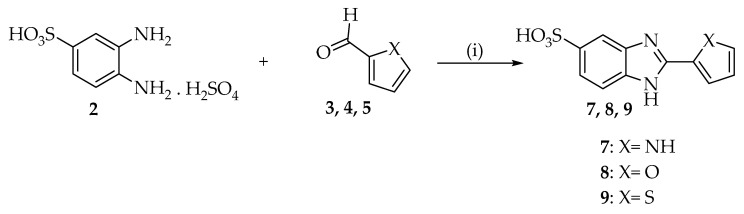
Synthesis of **7–9** 2-substituted benzimidazole-5-sulfonic acid. Reagents and conditions: (i) EtOH, NaHSO_3_ in H_2_O, reflux.

**Scheme 3:**
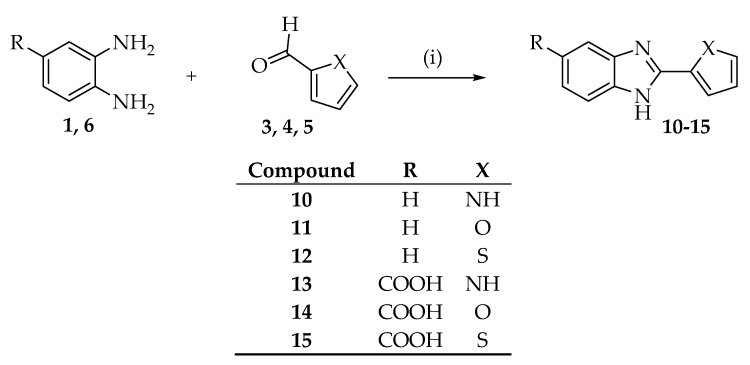
Synthesis of **10**–**15** benzimidazole derivatives. Reagents and conditions: (i) EtOH, NaHSO_3_ in H_2_O, reflux.

Benzimidazole derivatives were achieved by condensation reaction between *o*-phenylenediamine with aldehydes analogues as previously described [28]. In regard to 2-substituted benzimidazole-5-sulfonic acids (**7**–**9**), the intermediate 3,4-diaminobenzenesulfonic acid (**2**) was synthesized first. Compound **2** was easily obtained via Sulfonation reaction of *o*-phenylenediamine with sulfuric acid 96% (Scheme 1) and then, was condensed with aldehydes to obtain compounds **7**–**9** (Scheme 2). All reactions were carried out in the presence of NaHSO_3_ as oxidant agent. 2-substituted benzimidazoles (**10**–**12**) and 2-substituted benzimidazole-5-carboxilic acids (**13**–**15**) were directly obtained by addition of commercially available *o*-phenylenediamine (**1**) or 3,4-diaminobenzoic acid (**6**) and corresponding aldehydes (**3**–**5**) (Scheme 3).

The new and the already known synthesized compounds were divided into three classes (A–C) (Table S1 in Supplementary Materials). In each class, the compounds present different substituent at 5 position of the benzimidazole ring while the heterocycle in position 2 of the benzimidazole is the same.

### 2.2. Evaluation of Filtering Parameters

#### 2.2.1. In Vitro Photoprotective Activity of Benzimidazole Derivatives Solutions

The spectral behavior of the test compounds dissolved in methanol was investigated firstly because the solar protection is related to the UV absorption. Absorption spectra were measured from 290 to 400 nm. Compounds **10**, **11** and **12** have similar lambda max value, however lambda max of **12** is slightly higher than that of **11**, which in turn is slightly greater than that of **10**. Substitution of -H by withdrawing group (-COOH and –SO_3_H) at the 5 position on the benzimidazole ring shows the batochromic shift (data not shown). This long wavelength absorption band is associated with the intramolecular charge transfer transition due to the transfer of the charge between the five-membered rings to the electron-withdrawing of benzimidazole and neighboring carboxylic or sulfonic chromophore.

Sun Protection Factor (SPF) is the UV energy required for producing a minimal erythema dose (MED) on protected skin, divided by the UV energy required producing a MED on unprotected skin. This factor was first introduced in 1974 to evaluate the effectiveness of a sunscreen, only the in vivo method was officially accepted by the US Food and Drug Administration and COLIPA (now Cosmetics Europe—The Personal Care Association). Due to the high cost, time consumption and some questions noble of the volunteers relating to in vivo SPF determination, in vitro photoprotection studies have been developed. The broadly applied in vitro method is the well-known Diffey-Robson approach [29] that is a spectrophotometrically-based measurement of transmission. Spectral data of samples were collected from 290–400 nm with the calculator software [30]. The software automatically determines theoretical COLIPA—SPF, COLIPA—UVAPF, critical wavelength value (λc) and UVA/UVB ratio as preliminary data.

Photoprotective activity of the sunscreen against UVB is reflected by SPF value. According to the results (Table S2), SPF values of all synthesized compounds was high compared to lead compound (PBSA) and the highest SPF value belongs to compound **11** characterized by the presence of furan in position 2 of the benzimidazole ring and without functional group in position 5. By maintaining -SO_3_H group in position 5 and substituting the phenyl by the pyrrole, SPF value increased. However, by maintaining the pyrrole and replacing -SO_3_H with -COOH (**7** vs. **13**), SPF again increased. Then, substituting the functional group carboxyl by an -H (**13** vs. **10**), SPF exponentially increased. The same results were obtained by replacing phenyl with thiophene (**PBSA** vs. **9**, **12**, **15)** and furan **(PBSA** vs. **8**, **11**, **14)**. So, the photoprotective activity against the UVB radiations of the synthesized compounds does not depend only on the substitution in the 2-position of the benzimidazole ring but also on the groups present in position 5. Therefore the structure activity relationship is: -H > -COOH > -SO_3_H.

Moreover, replacing the pyrrole with the furan (**10** vs. **11**), the SPF value increased. On the other hand, by substituting the pyrrole with thiophene, the SPF decreased, for example **10** vs. **12** (SPF value of 13.13 to 7.03 respectively). Thus, by keeping the group present in the 5-position of benzimidazole and by varying the 5-membered ring at 2-position, the order of protection against UVB is as follows—furan > pyrrole > thiophene.

Protection from UVA rays is given by UVA-PF (UVA protection factor) which is considered effective if it is greater than or equal to 1/3 of SPF. Considering the data obtained, all the synthesized compounds showed UVA-PF values higher than those of the reference compound, while all remaining below 1/3 of the SPF. Therefore, the synthesized compounds are not potential candidates for UVA protection.

Critical wavelength is the parameter that provides information on the broad-spectrum of UV-filter. It is classified in five numerical categories: 0 (λc < 325 nm), 1 (325 ≤ λc ≤ 335), 2 (335 ≤ λc ≤ 350), 3 (350 ≤ λc <370) and 4 (λc ≥ 370). FDA considers the broad-spectrum sunscreen product should have a λc ≥ 370 nm [31]. Based on this classification, none of the synthesized compounds showed a value of λc ≥ 370 so they do not have a broad-spectrum profile.

In short, modifications on PBSA in position 2 by 5-membered rings and in position 5 by -COOH and -H led to enhanced UVB-filter activity.

#### 2.2.2. In Vitro Photoprotective Activity of Cosmetic Formulation Containing Benzimidazole Derivatives

Among synthesized compounds, it has been chosen to formulate the compounds **10**, **11** and **12**, since they are the ones that have shown the best SPF value in solution of their class and moreover all three are soluble in the same solvent which is PEG-7 Glyceryl Cocoate. The photoprotective efficacy of the synthesized compounds was determined by spectrophotometric measurements using the modified Diffey-Robson method [29]. The compounds were introduced at different concentrations (1%, 2% and 3%) in a 40:60 water-in-oil based cosmetic formulation and the analyzes were performed on PMMA (Poly(methyl methacrylate)) plates. 2-Phenyl-1H-benzo[d]imidazole-5-sulfonic acid (**PBSA**) was used as lead compound. Values of the SPF, UVAPF, UVA/UVB and λc are shown in Table 1. In comparison with lead compound, only compound **11**, among the selected synthesized compounds, provided higher protection against UVB radiation. **PBSA** at concentrations 1, 2 and 3% (w/w) showed increasing SPF values ranging from 3.22 to 5.09; while compound **11** which is benzimidazole with furan at the 2-position and unsubstituted at the 5-positon exhibited SPF values ranged from 6.88 to 11.15. Moreover replacing the furan with the pyrrole (**11** vs. **10**), the SPF value is decreased. On the other hand, by substituting the furan with thiophene **(11** vs. **12)**, the SPF also is decreased. However, compound **10** was slightly more UVB-protective than compound **12.**

Taking together the results obtained from selected benzimidazole derivatives, we could be deduced that the order of protection against UVB is as follows—furan-> pyrrole- > thiophene-derivatives.

#### 2.2.3. Photostability Study by Spectral Analysis

**Figure 2:**
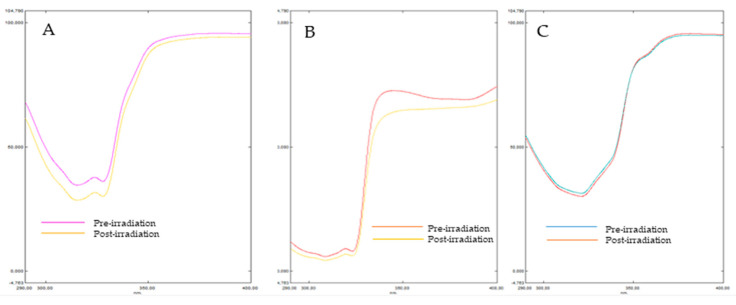
Transmittance profiles of formulations studied containing the benzimidazole derivatives **10** (panel **A**), **11** (panel **B**) and **12** (panel **C**) (1% *w*/*w*).

Photostability, which is one of the important parameters for defining the efficacy and safety of sunscreen, has been evaluated. So photodegradation was monitored by the UV transmittance measurements of the products before and after irradiation according to the criteria developed by Garoli et al. [32]. PMMA plates coated with sunscreen have been irradiated with the solar stimulator by the application of UVA dose necessary to cause erythema. Then, degree of photostability was expressed as the percentage effectiveness after exposure. Therefore in vitro % SPF_eff_ and %UVA-PF_eff_ were calculated according to Equations (4) and (5), respectively (paragraph 3.4.1). Obtained data (Figure 2) showed that the compounds **10** and **12** were photostable, since according to the regulation, sunscreen is considered photostable if the in vitro % SPF and % PF-UVA values are at least 80% [33]. In particular, compound **12**, bearing a thiophene in position 2, showed excellent photostability in the total UV range. However, compound **10** presenting pyrrole in position 2 was more photostable in UVA range than UVB range. Compounds **11** bearing furan at the 2-position was photoinstable with % SPF and% PF-UVA less than 80%. This is likely due to the insufficient photostability of its conjugated species [34].

Therefore the order of photostability of compounds is—thiophene derivative > pyrrole derivative > furan derivative.

### 2.3. Antioxidant Activity

The synthesized compounds were evaluated for their ability to scavenger the stable radical 2,2-diphenyl-1-picrylhydrazyl (DPPH) through DPPH test and their ability to reduced ferric ions to ferrous ions via Ferric reducing antioxidant power (FRAP) test.

From the results reported in Table 2, the reference compound is almost devoid of any antioxidant activity and the substitution of phenyl in the 2- position of benzimidazole by 5-membered rings gave the molecule an antioxidant activity more or less significant. Regarding DPPH test, compounds **7**, **10** and **14** showed percentage of inhibition greater than or equal to 50%. To this effect, they were screened to determine their IC_50_ value. However, only compound **10** exhibited good scavenger profile with IC_50_ = 64.098 µg/mL. Data obtained from FRAP showed that compounds **10** and **11**, respectively characterized by the presence of pyrrole and furan at the 2-position of the benzimidazole ring and both lacking a functional group at the 5-position showed the good antioxidant profile. Whereas compound **11** displayed the best activity (4462.64 μmolTE/g).

### 2.4. Antifungal Activity

Synthesized compounds were evaluated for their in vitro antifungal activity against five pathogenic dermatophytes—*Microsporum gypseum*, *Microsporum canis*, *Trichophyton mentagrophytes*, *Trichophyton tonsurans* and *Epidermophyton floccosum*. Percentage inhibition of fungal growth was performed using Sabouraud Dextrose Agar (SDA) as culture medium and DMSO as a solvent control. Incubation was done at room temperature and inhibition of the dermatophytes was evaluated daily by measuring the diameter of the colony at each disk over 7 days. All compounds were tested at the same concentration, namely 100 µg/mL and the results determined as the average of three different experiments were shown in Table S3.

Benzimidazole compounds unsubstituted in position 5 (compounds **10**, **11** and **12**) displayed strong growth inhibition of all five dermathophytes with percentage of inhibition closer to 100%. The presence of -SO_3_H at the 5-position decreased activity, however **7** and **9** showed good activity with an inhibition range of 50–79%. Curiously, compounds **13**, **14** and **15** bearing –COOH did not have a significant activity on dermatophytes; on the contrary, they rather favored the growth of certain fungi.

Interestingly, starting from **PBSA** which has no activity on dermatophytes and replacing phenyl by the five-membered cycle we obtained good results, indicating that the five-membered cycle is very important for the studied activity. Compounds **10**, **11** and **12** displayed fungicide activity on all five dermatophytes at 100 μg/mL. In addition, the substitution of pyrrole with thiophene or furan did not have influence on the variability of the activity; this is confirmed by Friedman which states that the bioisosteres have similar biological activity [35].

The structure-activity relationship reveals that the presence of hydrogen at the 5-position of benzimidazole ring is more beneficial than the electron withdrawing group.

Encouraged by these preliminary findings, compounds with growth inhibition close to 100% were further investigated by the determination of IC_50_. Thus, IC_50_ results of **10**, **11** and **12** against five dermatophytes are reported in Table 3. Fluconazole and Econazole nitrate were used as references agents. Tested compounds presented excellent activity toward *M. gypseum*, *M. canis, T. mentagrophytes*, *T. tonsurans* and *E. floccosum* with IC_50_ values between 0.97 and 3.80 µg/mL. Furthermore all tested compounds are more active than Fluconazole, except on *E. floccosum.* However, they are less effective than Econazole nitrate. Compound **10** exhibited the best activity, with IC_50_ values ranging from 0.97 to 1.53 μg/mL.

*Candida* is a fungus strain that can infect many parts of the human body. Among these, we can mention the infection of the skin. For this, we have also focused our attention on *Candida albicans* who lives in principle in the oral cavity, digestive tract, vagina and skin [36].

The tested compounds were dissolved in DMSO to obtain 12.8 mg/mL of stock solutions and the concentration tested ranged from 0.25 µg/mL to 128 µg/mL. The minimal inhibitory concentration (MIC) of the synthesized agents was determined by broth microdilution method using RPMI 1640 + MOPS as culture medium. After incubation in RPMI-160 broth at 37 °C for 24 and 48 h, the MIC (µg/mL) values were collected and reported on Table 4. Fluconazole was used as positive control drug. Compounds **7**, **9**, **14** and **15** showed activity against *Candida albicans* but, only **14** and **15** exhibited good activity (MIC = 16 µg/mL) after 24 h. These two compounds bearing carboxylic acid at the position 5 of benzimidazole ring. Neither synthesized compounds nor fluconazole showed activity after 48 h.

It is important to note that, contrary to the results observed on dermatophytes, the non-substituted compounds in position 5 of benzimidazole have no activity on the yeasts. This can be explained by the fact that each fungus has its own morphology and defense systems.

### 2.5. Antiproliferative Activity

In vitro antiproliferative activity of benzimidazole and benzothiazole derivatives against human melanoma cells (SK-Mel 5) was evaluated. Since benzimidazoles are recognized as potential anticancer drugs [10], we have also decided to examine the antiproliferative activity on other human cancer cells, including human T-lymphocyte cells (CEM), human cervical carcinoma cells (HeLa) and human pancreatic carcinoma cells (Mia Paca-2). Compound concentrations required to inhibit cell proliferation by 50% (in µM) are reported in Table 5. Non-cancer cells HEK293T (Normal Kidney Epithelial Cells) were chosen as a control. According to the obtained results, compound **10**, bearing pyrrole and unsubstituted at the 5-position, exhibited good inhibition against CEM, Mia Paca-2 and SK- MEL-5. Furthermore, compound **10** was 3-fold more selective against SK-Mel 5 (SI = 3.20) with IC_50_ = 9.7 µM against this tumor cell line.

### 2.6. Antiviral Activity

All the synthesized compounds were finally tested against herpes simplex virus-1 (KOS), herpes simplex virus-2 (G), herpes simplex virus-1 TK-KOS ACVr, vaccinia virus, Adeno virus-2 and Human Coronavirus (229E) in HEL cell cultures and against vesicular stomatitis virus, Coxsackie virus B4, Respiratory syncytial virus in HeLa cell cultures. Results are shown in Tables S4 and S5. None of the synthesized compounds showed specific antiviral activity.

## 3. Materials and Methods

### 3.1. General Information

All reagents were obtained from commercial sources and used without further purification. Analytical TLC was performed on silica gel plates (Macherey-Nagel Poligram SIL G/UV254 0.20 mm, GmbH & Co. KG Neumann-Neander-Str. 6–8, 52355 Dueren, Germany) and visualized by exposure to ultraviolet light (254 nm) and /or used 1% solution of KMnO_4_. Column chromatography was performed using silica gel Macherey-Nagel 60M 230400 mesh. ^1^H-NMR and ^13^C-NMR were registered on VXR-200 Varian spectrometer at 200 MHz and 400 MHz with tetramethylsilane (TMS) as an internal standard. Chemical shifts are reported in δ units (ppm) relative to the residual deuterated solvent signals of D_2_O and (CD_3_)_2_SO. The following abbreviations are used to indicate signal multiplicity and assignment—s (singlet), d (doublet), t (triplet), q (quartet), m (multiplet), brs (broad singlet) and dd (doublet of doublets), BIM (benzimidazole). Molecular weights of the compounds were determined by ESI (Micromass ZMD 2000) and the values are expressed as [MH]^+^. IR spectra were obtained using a Spectrum 100 FT-IR Spectrometer (PerkinElmer). UV spectrophotometric analyses were carried out on a UV-VIS spectrophotometer (Shimadzu UV-2600) or on a Life Science UV/VIS spectrophotometer (Beckman Coulter™, DU^®^530, Single Cell Module, Beckman Coulter s.r.l., Via Roma, 108 - Palazzo F1, Centro Cassina Plaza 20060 - Cassina De’ Pecchi, Milano, Italia). Photostability studies were performed with Atlas Suntest CPS+ solar simulator, (URAI S.p.a., Assago, Milano, Italy). Melting points were measured with a Stuart melting point apparatus. WW5 PMMA plates have been purchased from Schonberg GmbH (Munich, Germany). Sabouraud dextrose agar (SDA) was purchased by Sigma-Aldrich SRL, Milano, Italy.

### 3.2. Chemistry

#### 3.2.1. Synthesis of 3,4-diamino-benzensulfonic acid, Sulfate Salt (**2**)

To 0.5 g (4.62 mmoles) of *o*-phenylenediamine was added 3 mL of 96% sulfuric acid at 0 °C under stirring, then the solution was refluxed for 24 h. The reaction mixture was cooled and then cold water was added to obtain a precipitate. The mixture was filtered and washed with methanol to obtain 0.62 g of white powder, 46% yield. ^1^H NMR (400 MHz, DMSO-d6): δ(ppm) 7.39 (d, *J* = 1.6 Hz, 1H), 7.2 (dd, *J* = 1.6 Hz), 2H). ^13^C-NMR (400 MHz, DMSO-d6): 140.03, 138.39, 135.04, 118.22, 117.01.ESI-M [M + H] ^+^: calcd for C_6_H_8_N_2_O_3_S, 188.03; found 188.07.

#### 3.2.2. Synthesis of 2-substituted benzimidazole-5-sulfonic Acids

To a solution mixture of 3 mmol of **2** and 4 mmol of aldehyde (**3**–**5**) in ethanol (3 mL), 6 mmol of sodium bisulfite were added under stirring. The reaction mixture was heated at 80 °C under reflux for 24 h. The solvent was then evaporated under vacuum and the solid was treated with HCl 2N. The suspension was filtered and the solid was washed with ethanol to afford desired product.

##### 2-(1H-pyrrol-2-yl)-1H-benzimidazole-5-sulfonic Acid (**7**)

Brown solid in yield 56%; m.p. > 250 °C. IR (KBr) cm^−1^: 2851.26, 1638.62, 1456.40, 1227.98, 1143.18, 1023.50, 718.04. ^1^HNMR (400 MHz, DMSO-d6): δ (ppm) 11.89 (s, 1H, NH), 7.91 (s, 1H, BIM), 7.77–7.69 (m, 2H, BIM), 7.45 (d, *J* = 1.6 Hz, 1H, pyrrole), 7.30 (d, *J* = 1.6 Hz, 1H, pyrrole), 6.45 (t, 1H, pyrrole). ^13^C-NMR (400 MHz, DMSO-d6): 145.68, 143.67, 131.10, 130.45, 126.90, 123.22, 115.98, 115.04, 112.36, 111.36, 110.30. ESI-M [M + H] ^+^: calcd for C_11_H_9_N_3_O_3_S, 263.04; found 263.17.

##### 2-(furan-2-yl)-1H-benzimidazole-5-sulfonic Acid (**8**)

White solid in yield 31%; m.p. > 250 °C. IR (KBr) cm^−1^: 3446.68, 1589.75, 1474.34, 1316.39, 1217.18, 756.85.^1^HNMR (400 MHz, DMSO-d6): δ (ppm) 8.25 (d, *J* = 1.7 Hz, 1H, furan), 7.90 (d, *J* = 1.3 Hz, 1H, BIM), 7.78–7.68 (m, 2H, BIM), 7.60 (d, *J* = 3.6 Hz, 1H, furan), 6.93 (dd, *J* = 3.6 Hz, 1H, furan). ^13^C-NMR (100 MHz, DMSO-d6): δ (ppm) 147.90, 146.05, 141.44, 140.08, 132.89, 132.02, 123.40, 116.19, 13.47, 110.94. ESI-M [M + H] ^+^: calcd for C_11_H_8_N_2_O_4_S, 264.02; found 264.22.

##### 2-(thiophen-2-yl)-1H-benzimidazole-5-sulfonic Acid (**9**)

White solid in yield 69%; m.p. > 250 °C. IR (KBr) cm^−1^: 2645.75, 1631.52, 1458.42, 1079.51, 1026.70, 718.69. ^1^HNMR (400 MHz, DMSO-d6): δ (ppm) 8.14 (d, *J* = 1.6 Hz, 1H, thiophene), 8.11 (d, *J* = 1.6 Hz, 1H, thiophene), 7.85 (s, 1H, BIM), 7.71–7.62 (m, 2H, BIM), 7.39 (t, 1H, thiophene). ^13^C-NMR (400 MHz, DMSO-d6): 145.45, 143.42, 141.67, 140.39, 128.00, 121.88, 116.43, 113.09. ESI-M [M + H] ^+^: calcd for C_11_H_8_N_2_O_4_S_2_, 280.00; found 280.18.

#### 3.2.3. General Method for the Synthesis of 2-Substituted Benzimidazoles

To a solution mixture of 3 mmol of **1** and 4 mmol of aldehyde (**3**–**5**) in ethanol (3 mL), 6 mmol of sodium bisulfite were added under stirring. The reaction mixture was heated at 80 °C under reflux for 24 h. The solvent was then evaporated under vacuum and the solid was treated with HCl 2N. The suspension was filtered and the solid was washed with ethanol to afford desired product.

##### 2-(1H-pyrrol-2-yl)-1H-benzimidazole (**10**)

Yellow solid in yield 55%; m.p. > 250 °C. IR (KBr) cm^−1^: 2733.42, 1606.25, 1464.35, 1227.52, 1136.01, 1041.46, 743.84. ^1^HNMR (400 MHz, DMSO-d6): δ (ppm) 11.99 (brs, 1H, NH pyrrole), 7.65–7.62 (m, 2H, BIM), 7.34–7.29 (m, 2H, BIM), 7.19 (d, *J* = 1.6 Hz, 1H, pyrrole), 7.11 (d, *J* = 1.4 Hz, 1H, pyrrole), 6.33 (t, 1H, pyrrole). ^13^C-NMR (400 MHz, DMSO-d6): 154.34, 134.04, 120.89, 119.30, 113.45, 110.11, 105.86.ESI-M [M + H] ^+^: calcd for C_11_H_9_N_3_, 183.08; found 183.12.

##### 2-(furan-2-yl)-1H-benzimidazole (**11**)

Yellow solid in yield 60%; m.p. > 250 °C. IR (KBr) cm^−1^: 2619.65, 1569.95, 1419.27, 1234.04, 1073.76, 850.94, 740.26. ^1^HNMR (400 MHz, DMSO-d6): δ (ppm) 8.10–7.90 (d, *J* = 1.7 Hz, 1H, furan), 7.19 (t, 2H, BIM), 7.09 (m, 3H, 2H BIM, 1H furan), 6.70 (t, 1H, furan)_._^13^C-NMR (400 MHz, DMSO-d6): 154.04, 141.45, 140.76, 139.11, 123.45, 115.09, 112.86, 107.23. ESI-M [M + H] ^+^: calcd for C_11_H_8_N_2_O, 184.06; found 184.21.

##### 2-(thiophen-2-yl)-1H-benzimidazole (**12**)

Grey Solid in yield 72%; m.p. > 250 °C. IR (KBr) cm^−1^: 2619.65, 1569.95, 1449.98, 1419.27, 1234.04, 850.94, 702.14. ^1^HNMR (400 MHz, DMSO-d6): δ (ppm) 7.98–7.72 (m, 4H, 2H BIM, 2H thiophene), 7.24 (t, *J* = 1.5 Hz, 2H, BIM), 7.00 (t, 1H, thiophene). ^13^C-NMR (400 MHz, DMSO-d6): 145.33, 142.71, 140.11, 139.67, 128.07, 127.23, 126.61, 122.00, 114,25. ESI-M [M + H] ^+^: calcd for C_11_H_8_N_2_S, 200.04; found 200.11.

#### 3.2.4. General Method for the Synthesis of 2-Substituted Benzimidazole-5-Carboxylic Acids

To a solution mixture of 3 mmol of **6** and 4 mmol of aldehyde (**3**–**5**) in ethanol (3 mL), 6 mmol of sodium bisulfite were added under stirring. The reaction mixture was heated at 80 °C under reflux for 24 h. The solvent was then evaporated under vacuo and the solid was treated with HCl 2N. The suspension was filtered and the solid was washed with ethanol to afford desired product.

##### 2-(1H-pyrrol-2-yl)-1H-benzimidazole -5-carboxylic Acid (**13**)

Brown solid in yield 51%; m.p. > 250 °C. IR (KBr) cm^−1^: 2678.85, 1624.06, 1600.08, 1459.86, 1302.43, 1137.71, 896.74, 763.89. ^1^H NMR (400 MHz, DMSO-d6): δ (ppm) 11.23 (brs, 1H, OH), 8.14 (s, 1H, BIM), 7.94–7.80 (m, 2H, BIM), 6.77(d, *J* = 1.5 Hz, 1H, pyrrole), 6.50 (d, *J* = 1.5 Hz, 1H, pyrrole), 6.23 (t, 1H, pyrrole). ^13^C-NMR (400 MHz, DMSO-d6): 166.00, 155.68, 146.91, 138.56, 128.87, 126.88, 120.55, 116.31, 111.75, 107.70. ESI-M [M + H] ^+^: calcd for C_12_H_9_N_3_O_2_, 227.07; found227.32.

##### 2-(furan-2-yl)-1H-benzimidazole -5-carboxylic Acid (**14**)

Brown solid in yield 49%; m.p. > 250 °C. IR (KBr) cm^−1^: 2500.23, 1686.09, 1538.50, 1290.52, 1234.76, 104.40, 766.43. ^1^HNMR (400 MHz, DMSO-d6): δ (ppm) 11.19 (brs, 1H, OH), 8.30 (d, *J* = 1.6 Hz, 1H, furan), 8.16 (s, 1H, BIM), 8.06 (d, *J* = 1.6 Hz, 1H, BIM), 7.89 (d, *J* = 1.5 Hz, 1H, BIM), 7.76 (d, *J* = 3.6 Hz, 1H, furan), 6.50 (t, 1H, furan). ^13^C-NMR (400 MHz, DMSO-d6): 166.73, 154.02, 145.55, 142.03, 139.40, 138.00, 126.62, 120.13, 115.74, 113.11, 106.85. ESI-M [M + H] ^+^: calcd for C_12_H_8_N_2_O_3_, 228.05; found 228.14.

##### 2-(thiophen-2-yl)-1H-benzimidazole -5-carboxylic Acid (**15**)

Grey solid in yield 69%; m.p. > 250 °C. IR (KBr) cm^−1^: 2490.11, 1682.64, 1581.93, 1416.22, 1286.30, 854.62, 768.66. ^1^HNMR (400 MHz, DMSO-d6): δ (ppm) 11.26 (brs, 1H, OH), 8.25 (s, 1H, BIM), 8.02 (d, *J* = 1.6 Hz, 1H, BIM), 7.82–7.75 (m, 3H, 1H BIM, 2H thiophene), 7.01 (t, 1H, thiophene). ^13^C-NMR (400 MHz, DMSO-d6): 166.07, 146.76, 141.86, 138.62, 129.11, 118.65, 114.04, 113.29. ESI-M [M + H] ^+^: calcd for C_12_H_8_N_2_O_2_S_2_, 244.03; found 244.26.

### 3.3. Biological Assays

#### 3.3.1. In Vitro Photoprotection Activity

##### Filtering Parameters Evaluation of Benzimidazole Derivatives in Solution

The assessment of Sun protection factor was performed using a SHIMADZU UV-2600 UV–VIS spectrophotometer (Shimadzu Italia S.r.l., Via G.B. Cassinis 7, 20139 Milano, Italy). Test compounds were dissolved in MeOH at the concentration of 0.0015 (±0.000005) % and the absorbance was measured between 290 and 400 nm using a 1 cm quartz cell at intervals of one 1 nm using a UV–Vis Spectrophotometer. The absorbance at wavelength λ is related to the transmittance (T(λ)) by the Equation (1).

A(λ) = Log [T(λ)],(1)

where T(λ) is the fraction of incident irradiance transmitted by the sample.

##### Sunscreens Formulation

A basic topical formulation W/O was prepared—hydrophilic-phase and oil-phase were heated separately to between 60–70 °C, until complete solubilization of the ingredients. Subsequently, the hydrophilic phase was slowly added to the oil phase under constant stirring with a Silverson homogenizer. Then, the emulsion was cooled to room temperature and the selected sunscreen agents were incorporated at different concentrations (1%, 2% and 3%).

INCI—aqua, glycerin, Euxyl PE 9010, Xanthan gum, triethanolamine, ceteareth-12, ceteareth-20, Sterearic acid, Butylhydroxytoluene, Myritol, PEG-7 glycerylcocoate, sodium hydroxide.

##### In Vitro Evaluation of Filtering Parameters

32.5 mg of each formulation were spread onto 25 cm^2^ PMMA plates with finger. Plates were weighed before and after spreading. Three plates were prepared for each product and five measurements were performed on each plate. The plates were left at room temperature in the dark for 15 to 30 min before measurements. Then, the UV transmittance measurements were carried out from 290 to 400 nm using UV-VIS spectrophotometer (Shimadzu UV-2600). The blank was prepared using a plate covered with 15 µL of glycerin thanks to its non-fluorescent and UV transparency. In vitro SPF, UVA-PF and Critical Wavelength were carried out according using SPF Calculator Software (SPF Calculator Software (version 2.1), Shimadzu, Milan, Italy) as previously described [37].

#### 3.3.2. Antioxidant Activity

##### DPPH Assay

The activity against DPPH• radical was tested following the Wang et al. method [38] modified as previously reported [39]. The synthesized derivatives were initially tested at the same concentration of 1 mg/mL to determine a result expressed as μmol TE/g. Subsequently, the better profile compounds were tested against the radical to determine the IC_50_ value expressed as µg/mL.

##### FRAP Assay

The FRAP antioxidant capacity of synthesized compounds was analyzed according as previously described [40], measuring the absorbance of the reaction mixture at 593 nm. The values are expressed as μmol TE/g compound.

#### 3.3.3. Antifungal Activity

##### Antidermatophyte Activity

Microorganism

Dermatophytes used in this study were *Epidermophyton floccosum* var. floccosum (Netherlands) CBS 358.93 strain; *Trichophyton tonsurans* (Netherlands) CBS 483.76 strain; *Trichophyton mentagrophytes* (Netherlands) CBS 160.66 strain; *Microsporum canis* (Iran) CBS 131110 strain; and *Microsporum gypseum* (Iran) CBS 130948 strain. All dermatophytes were maintained at 4 °C as agar slants on SDA.

In Vitro Culture

In Vitro antifungal activity against the five dermatophytes was assessed using the plaque growth inhibition method as previously described [37]. Each compound was evaluated at the final concentration of 100 µg/mL. Percentages of growth inhibition were calculated by comparing mean value of diameters of the mycelia in test plates with that of untreated control plates following the Equation (2). The percent inhibition of growth was determined as the average of three different experiments.

I = [(C − T)/C] × 100%,(2)

where, I is the growth inhibition rate (%), C is the extended diameter of the circle mycelium of the control (mm) and T is the extended diameter of the circle during testing (mm). IC_50_ values were obtained only for the benzimidazole that were active against the five dermatophytes. The IC_50_ values were calculated as the average of three different experiments and they indicate the concentration of the substance needed to inhibit the growth of the fungus by half.

##### Anti-*Candida albicans* Activity

Fungal suspension was prepared by suspending a *Candida albicans* (ATCC 10231) aliquot in 5 mL of sterilized water. Stock solutions were prepared by dissolving the compounds and Fluconazole in DMSO at a concentration of 12.80 mg/mL. Minimal inhibitory concentration (MIC) of the synthesized agents was performed by broth microdilution method according to the Clinical and Laboratory Standards Institute/National Committee for Clinical Laboratory Standards (CLSI/NCCLS) Approved Standard M27-A3, 2008 [NCCLS] as previously described [37].

#### 3.3.4. Anti-Proliferative Activity

##### Cell Lines

Human cervical carcinoma (HeLa) and human CD_4_^+^T-lymphoblast (CEM) cells were obtained from ATCC (Middlesex, UK). Human pancreatic carcinoma (Mia-Paca 2) cells were kindly provided by Prof. Anna Karlsson (Karolinska Institute, Stockholm, Sweden). All cell lines were grown in Dulbecco’s modified Eagle’s medium (DMEM; Gibco, Carlsbad, CA, USA), supplemented with 10% fetal bovine serum (FBS, Gibso), 0.01M Hepes (Gibco) and 1 mM sodium pyruvate (Gibco) in a humidified 5% CO_2_ incubator at 37 °C.

##### Cell Proliferation

Suspension of CEM cells were seeded in 96-well microtiter plates at 60,000 cells/well in the presence of different concentrations of the compounds. The cells were allowed to proliferate for 48 h or 72 h, respectively and then counted in a Coulter counter. The 50% inhibitory concentration (IC_50_) was defined as the compound concentration required to reduce cell proliferation by 50%. HeLa and Mia-Paca2 cells were seeded in 96-well plates at 15,000 cells/well in the presence of different concentrations of the compounds. After 4 days of incubation, the cells were trypsinized and counted in a Coulter counter).

##### IC_50_ Determination

The compounds were dissolved in DMSO at 20 mM (stock solution) and kept in the refrigerator until use. Then, compound dilutions were made in cell culture medium and serial compound concentrations were tested starting at 100 μM as the highest concentration. The IC_50_ values were calculated using Equation (3).

C1 − [50 − N1%/N2% − N1%] − (C1 − C2),(3)

wherein C1 is the compound concentration that inhibits cell proliferation more than 50%; C2 is the compound concentration that inhibits cell proliferation less than 50%; N1% represents the cell number (in percent of control in the absence of compound) obtained in the presence of C1 and N2% represents the cell number (in percent of control in the absence of compound) obtained in the presence of C2.

#### 3.3.5. Anti-Viral Activity

Antiviral activity was tested against herpes simplex virus-1 (KOS), herpes simplex virus-2 (G), herpes simplex virus-1 TK-KOS ACVr, vaccinia virus, Adeno virus-2 and Human Coronavirus (229E) in HEL cell cultures; vesicular stomatitis virus, Coxsackie virus B4, Respiratory syncytial virus in HeLa cell cultures. Cytotoxicity of compounds was evaluated in parallel with the antiviral activity namely, microscopic evaluation of the cell morphology of the confluent cell monolayer which either had or had not been inoculated with virus. Cytotoxic concentration and concentration producing 50% inhibition of virus-induced cytopathic effect were determined by measuring the cell viability with the colorimetric formazan-based MTS assay ((3-(4,5-dimethylthiazol-2-yl)-5-(3-carboxymethoxyphenyl)-2-(4-sulfophenyl)-2H-tetrazolium)). Reference compounds used were—Brivudin, Cidofovir, Ganciclovir, Acyclovir for virus in HEL cell cultures and ribavirin and DS-10.000 for virus in HeLa cell cultures. The antiviral activity of the compounds was expressed as the concentration required to inhibit viral cytopathogenicity by 50% (EC_50_).

### 3.4. Stability Studies

#### Photostability

The photostability was determined using the method previously described [37]. Each plate, prepared according to the previous section was irradiated with a solar simulator applying the UVA-dose equivalent to an effective erythema radiant exposure. The spectral transmittance of thin film of sunscreen was measured before and after sunlight exposure from 290 to 400 nm. The residual percentage of SPF in vitro (% SPF_eff._) and UVA-PF (% UVA-PF_eff._)) were calculated according to Equations (4) and (5) respectively. In fact, a filter is considered photostable if % SPF_eff._ and % UVA-PF_eff._ are more than or equal to 80.

%SPF_eff_. = in vitro SPF_after_/ in vitro SPF_before_ × 100 (4)

%UVA − PF_eff_. = UVA − PF_after_ /UVA-PF_before_ × 100. (5)

## 4. Conclusions

The isosteric modification approach has been exploited to design and investigate the structure-activity relationship of a series of some new and other already known nine benzimidazole derivates.

Regarding the antifungal activity, compounds **10**, **11** and **12** exhibited good activity against *M. gypseum*, *M. canis, T. mentagrophytes*, *T. tonsurans* and *E. floccosum* with IC_50_ values in the range of 0.97–3.80 µg/mL. Nevertheless, compound **10** showed the best activity with IC_50_ range of 0.97–1.53 µg/mL. This compound is characterized by the presence of pyrrole in position 2 of the benzimidazole ring and devoid functional group in position 5. In addition, compounds **14** and **15** exhibited good activity against *Candida albicans* with MIC = 16 µg/mL.

Considering UV-filtering capacity, compounds **10**, **11** and **12** showed to be a good UVB-filter as compared to PBSA and the best photoprotective compound was **11** bearing furan in position 2 of the benzimidazole ring and without substitution in position 5. Compounds **10** and **11** showed good antioxidant activity; however according to DPPH test only **10** was endowed with antioxidant activity, while according to FRAP test, both **10** and **11** had good antioxidant activity being **11** (4462.64 µmol TE/g) the best one.

Finally, regarding the antiproliferative activity, compounds **10** displayed good activity against SK-Mel 5 (human melanoma cells) with IC_50_ = 9.7 µM.

Taken together all performed biological test, the best multifunctional molecule was compound **10**, which well showed a wide range as UV-filter, antioxidant, antifungal and antiproliferative agent. Compound **11** also displayed good UV-filter, antioxidant and antifungal activities, although was not endowed of any antiproliferative activity. Thus, compound **10** is a potential candidate in the development of drugs in case of cutaneous neoplastic diseases, in particular melanoma. Therefore, this series of benzimidazole derivatives could be useful for developing novel multifunctional molecules for the treatment of multifactorial diseases.

## Figures and Tables

**Table 1:** UV filtering activity of benzimidazole derivatives **10**–**12** in formulation at different concentrations.

Compound	Percentage (%)	SPF	UVAPF	UVA/UVB	λc (nm)
	**1**	3.22	0.94	0.33	319
**PBSA**	**2**	4.63	0.94	0.22	321
	**3**	5.09	1.02	0.25	324
	**1**	2.44	1.16	0.70	341
**10**	**2**	2.72	1.32	0.73	357
	**3**	3.06	1.54	0.75	364
	**1**	6.84	1.94	0.69	368
**11**	**2**	10.39	1.53	0.13	366
	**3**	11.15	1.82	0.18	371
	**1**	2.41	1.16	0.71	340
**12**	**2**	2.59	1.22	0.70	342
	**3**	2.79	1.26	0.69	344

**Table 2:** Antioxidant activity of the compounds through DPPH and FRAP test. Each value was obtained from three different experiments (mean ± SEM). (The caffeic acid value of the DPPH test refers to a tested concentration equal to 0.0105 mg/mL).

Compound	DPPH (% Inhibition)	DPPH IC_50_ (µg/mL)	FRAP (µmolTE/g)
**Caffeic acid**	81.35 ± 0.35	-	10156.12 ± 15.5
**PBSA**	<LOQ *	-	<LOQ *
**7**	53.88 ± 2.30	1093.09 ± 8.65	239.91 ± 1.79
**10**	70.00 ± 3.25	64.10 ± 3.21	1085.57 ± 0.88
**13**	33.00 ± 1.28	-	108.89 ± 2.98
**8**	10.96 ± 0.56	-	<LOQ *
**11**	15.46 ± 0.98	-	4462.64 ± 18.29
**14**	52.06 ± 1.48	1416.38 ± 21.40	26.67 ± 2.89
**9**	12.52 ± 0.88	-	82.16 ± 0.22
**12**	35.05 ± 0.87	-	75.89 ± 5.43
**15**	30.23 ± 1.45	-	181.84 ± 2.66

* LOQ, Limit of Quantification.

**Table 3:** IC_50_ values of compounds **13**–**15**, Econazole nitrate and Fluconazole on dermatophytes. Each value is the mean of three measurements.

	IC_50_ (µg/mL)				
Compound	*M. gypseum*	*M. canis*	*T. mentagrophytes*	*T. tonsurans*	*E. floccosum*
**10**	1.53 ± 0.05	1.34 ± 0.02	1.38 ± 0.08	0.97 ± 0.06	1.07 ± 0.03
**11**	1.54 ± 0.02	1.58 ± 0.06	1.61 ± 0.10	1.89 ± 0.22	2.44 ± 0.05
**12**	1.55 ± 0.03	2.14 ± 0.09	1.74 ± 0.11	2.42 ± 0.15	3.80 ± 0.07
**Fluconazole**	18.5 ± 1.23	29.6 ± 1.84	3.53 ± 0.26	19.41 ± 0.87	0.08 ± 0.005
**Econazole nitrate**	0.05 ± 0.0001	0.51 ± 0.02	0.006 ± 0.0001	0.13 ± 0.008	0.47 ± 0.015

**Table 4:** Anti-*Candida albicans* activity of synthesized compounds. The results are representative of incubations arranged in triplicate at 24 and 48 h and the average values were taken as the minimal inhibitory concentrations (MICs).

	MIC (µg/mL)	
Compound	24 h	48 h
**7**	64	-
**10**	-	-
**13**	-	-
**8**	-	-
**11**	-	-
**14**	16	-
**9**	64	-
**12**	-	-
**15**	16	-
**Fluconazole**	0.5	-

**Table 5:** Inhibitory effects of benzimidazole derivatives on cell proliferation (CEM, HeLa, Mia Paca-2 and SK-Mel 5). All analyzes are the mean of triplicate measurements ± standard deviation.

	IC_50_ (µM)					SI *			
Compound	CEM	HeLa	Mia Paca-2	SK-Mel 5	Hek293	CEM	HeLa	Mia Paca-2	SK-Mel 5
**7**	31 ± 21	>100	28 ± 10	>100	67 ± 47	2.16	-	2.39	-
**10**	14 ± 1	37 ± 0	15 ± 9	9.7 ± 1.7	31 ± 3	2.21	-	2.07	3.20
**13**	>100	>100	>100	>100	>100	-	-	-	-
**8**	>100	≥100	≥100	≥100	>100	-	-	-	-
**11**	>100	>100	>100	>100	>100	-	-	-	-
**14**	75 ± 35	>100	>100	>100	>100	1.33	-	-	-
**9**	34 ± 2	>100	43 ± 14	>100	>100	2.94	-	2.33	-
**12**	>100	>100	≥100	86 ± 17	>100	-	-	-	1.16
**15**	50 ± 10	>100	80 ± 12	>100	>100	2	-	1.25	-

* SI = IC_50_ of pure compound in a normal cell line/IC50 of the same pure compound in cancer cell line.

## Supplementary Materials

The following are available online. Figure S1: Representative ^13^C-NMR spectrum of newly synthesized compound **8**, Figure S2: Representative ^1^H-NMR spectrum of newly synthesized compound **8,** Figure S3: Representative ^13^C-NMR spectrum of newly synthesized compound **7**, Figure S4: Representative ^1^H-NMR spectrum of newly synthesized compound **7,Table S1**: The three classes of benzimidazole derivatives, Table S2: UV filtering activity of benzimidazole derivatives **7**–**15** in solution, Table S3: Percent growth inhibition of dermatophytes treated with benzimidazole derivatives at 100 μg/mL. Each value is the mean of three measurements, Table S4: Cytotoxicity and antiviral activity of compounds in human embryonic lung (HEL) cell cultures, Table S5: Cytotoxicity and antiviral activity of compounds in HeLa cell cultures.
