# Supplementary material for: Skin Damages—Structure Activity Relationship of Benzimidazole Derivatives Bearing a 5-Membered Ring System
Source: Molecules. 2020 Sep 21;25(18):4324. doi: 10.3390/molecules25184324 (PMC7570844; doi:10.3390/molecules25184324)
Supplement: Supplementary file 1 [file molecules-25-04324-s001.pdf]

Skin damages: Structure Activity Relationship of benzimidazole derivatives bearing a 5-membered ring system

Ernestine Nicaise Djuidje [1], Elisa Durini [1], Sabrina Sciabica [2], Elena Serra [3], Jan Balzarini[4], Sandra Liekens [4], Stefano Manfredini [1], Silvia Vertuani [1], and Anna Baldisserotto [1,*]

Table S1. The three classes of benzimidazole derivatives.

**Class A**	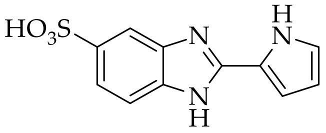 **7**	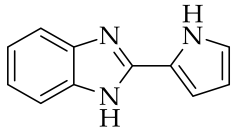 **10**	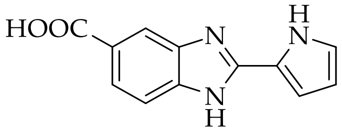 **13**
**Class B**	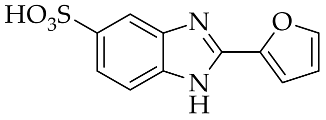 **8**	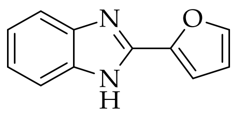 **11**	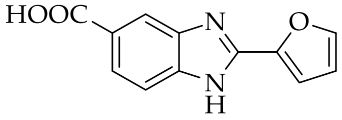 **14**
**Class C**	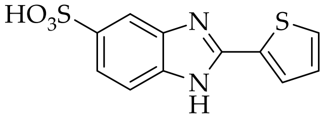 **9**	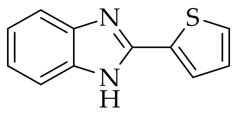 **12**	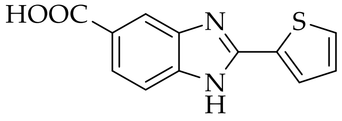 **15**

Table S2. UV filtering activity of benzimidazole derivatives **7-15** in solution.

Compound	SPF	UVAPF	UVA/UVB	λc (nm)
**PBSA**	3.40±0.17	1.03±0.08	0.29	322
**7**	5.43±0.39	1.22±0.03	0.2	345
**10**	13.13±0.70	1.16±0.05	0.02	325
**13**	7.40±0.23	1.10±0.09	0.7	345
**8**	10.96±0.54	1.33±0.05	0.29	342
**11**	20.06±3.04	1.05±0.06	0.3	333
**14**	16.66±1.21	1.20±0.10	0.5	332
**9**	4.72±0.17	1.43±0.09	0.48	339
**12**	7.03±0.42	1.49±0.07	0.37	345
**15**	6.40±0.12	1.42±0.11	0.43	342

Table S3. Percent growth inhibition of dermatophytes treated with benzimidazole derivatives at 100 μg/mL. Each value is the mean of three measurements.

	Percent growth inhibition				
**Compound (100 μg/mL)**	***M. gypseum***	***M. canis***	***T. mentagrophytes***	***T. tonsurans***	***E. floccosum***
**PBSA**	9.62 ± 0.61	+	+	+	7.41 ± 0.43
**7**	73.04 ± 3.67	72.11 ± 7.51	69.03 ± 1.84	56.58 ± 4.04	52.46 ± 6.58
**10**	99.07 ± 1.33	96.85 ± 3.56	96.26 ± 2.42	96.97 ± 0.62	101.75 ± 4.92
**13**	3.74 ± 1.49	8.66 ± 1.78	+	4.55 ± 1.34	21.05 ± 2.14
**8**	8.00 ± 2.44	+	+	+	+
**11**	99.00 ± 0.28	98.32 ± 2.03	97.22 ± 4.53	94.74 ± 5.63	97.87 ± 4.23
**14**	+	18.72 ± 0.59	+	+	6.45 ± 1.47
**9**	59.80 ± 6.12	65.91 ± 3.21	57.27 ± 1.09	50.75 ± 2.11	50.00 ± 3.26
**12**	100.00 ± 1.63	92.91 ± 1.33	98.13 ± 2.76	96.97 ± 1.34	94.74 ± 5.45
**15**	2.14 ± 0.59	20.30 ± 0.54	+	4.48 ± 0.90	6.45 ± 2.98

Table S4. Cytotoxicity and antiviral activity of compounds in human embryonic lung (HEL) cell cultures.

		Antiviral EC_{50}[b] (μM)					
**Compound**	**Cytotoxic concentration[a] (μM)**	**Herpes simplex virus-1 (KOS)**	**Herpes simplex virus-2**	**Herpes simplex virus-1 TK-KOS ACVr**	**Vaccinia virus**	**Adeno virus-2**	**Human Coronavirus (229E)**
**7**	>100	>100	>100	>100	>100	>100	>100
**10**	>100	>100	>100	>100	>100	>100	>100
**13**	>100	>100	>100	>100	>100	>100	>100
**8**	>100	>100	>100	>100	>100	>100	>100
**11**	>100	>100	>100	>100	>100	>100	>100
**14**	>100	>100	>100	>100	>100	>100	>100
**9**	>100	>100	>100	>100	>100	>100	>100
**12**	>100	>100	>100	>100	>100	>100	>100
**15**	>100	>100	>100	>100	>100	>100	>100
**Birivudin**	>250	0.01	250	0.1	5.8	-	-
**Cidofovir**	>250	4.5	3.4	2.8	50	10	-
**Acyclovir**	>250	0.6	0.6	2	>250	-	-
**Ganciclovir**	>250	0.01	0.01	0.2	>100	-	-

[a] Cytotoxic concentration, as determined by measuring the cell viability with the colorimetric formazan-based MTS assay. [b]concentration producing 50% inhibition of virus-induced cytopathic effect, as determined by MTS method. – not detected.

Table S5. Cytotoxicity and antiviral activity of compounds in HeLa cell cultures.

Compound	Cytotoxic concentration[a] (µM)	Antiviral EC_{50}[b] (µM)		
		Vesicular stomatitis virus	Coxsackie virus B4	Respiratory syncytial virus
**7**	>100	>100	>100	>100
**10**	>100	>100	>100	>100
**13**	>100	>100	>100	>100
**8**	>100	>100	>100	>100
**11**	>100	>100	>100	>100
**14**	>100	>100	>100	>100
**9**	>100	>100	>100	>100
**12**	>100	>100	>100	>100
**15**	>100	>100	>100	>100
**DS-10.000**	>100	>100	>100	0.8
**Ribaravin**	>250	112	250	10

[a] Cytotoxic concentration, as determined by measuring the cell viability with the colorimetric formazan-based MTS assay. [b]concentration producing 50% inhibition of virus-induced cytopathic effect, as determined by MTS method.

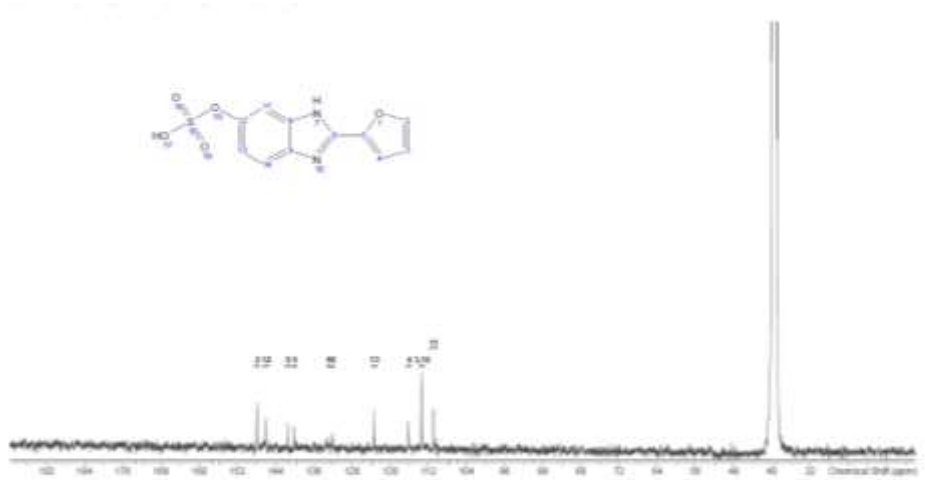

Figure S1. Representative ^{13}C-NMR spectrum of newly synthesized compound **8**.

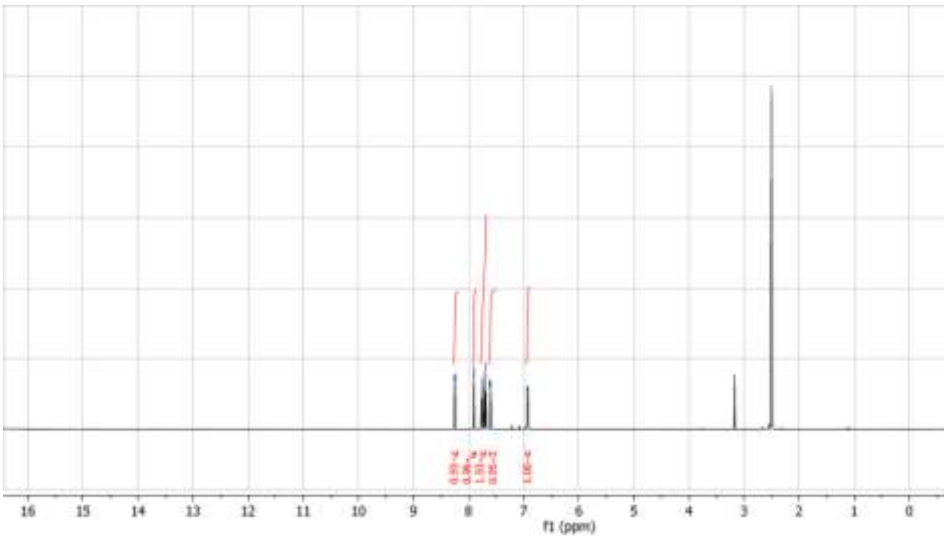

Figure S2. Representative ^{1}H-NMR spectrum of newly synthesized compound **8**.

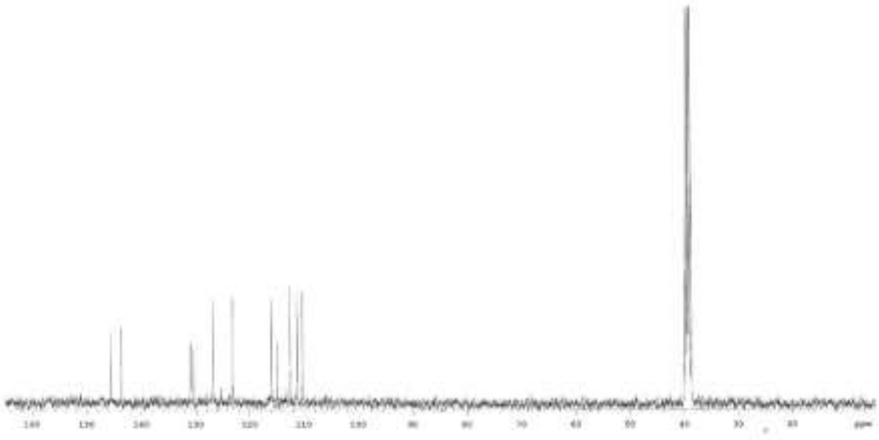

Figure S3. Representative ^{13}C-NMR spectrum of newly synthesized compound **7**.

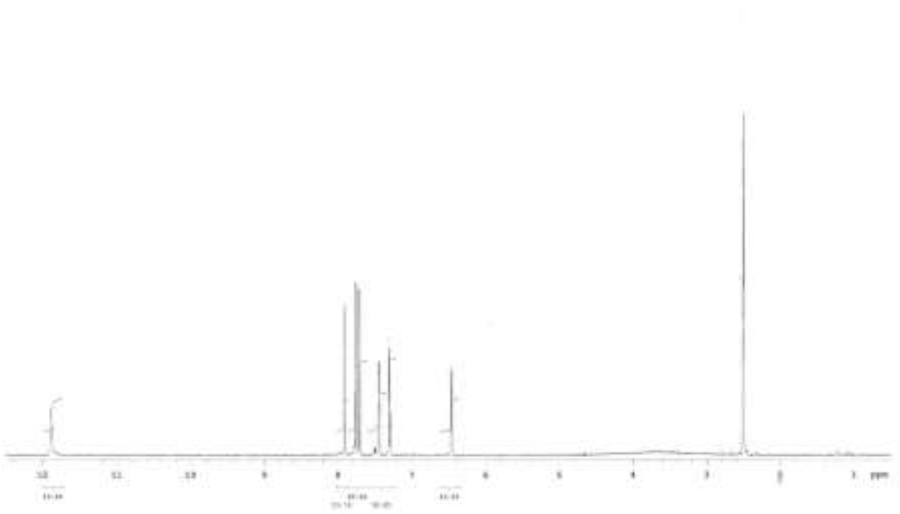

Figure S42. Representative ^{1}H-NMR spectrum of newly synthesized compound **7**.

Following the general procedure benzimidazoles **9** [CAS number 1158706-59-2], **10** [1S], **11** [1S], **12** [2S, 3S], **13** [**CAS Registry Number** 1030658-13-9], **14** [4S], and **15** [**CAS Registry Number** 1158380-17-6, 5S] were prepared and their analytical and spectral data are in agreement with those reported in literature or those reported as commercial sources.

References:

1S. Senapak, W.; Saeeng, R.; Jaratjaroonphong, J.; Promarak, V.; Sirion, U. Metal-free selective synthesis of 2-substituted benzimidazoles catalyzed by Bronsted acidic ionic liquid: Convenient access to one-pot synthesis of N-alkylated 1,2-disubstituted benzimidazoles. *Tetrahedron* **2019**, *75*, 3543-3552.

2S. Das, K.; Mondal, A.; Srimani, D. Selective Synthesis of 2-Substituted and 1,2-Disubstituted Benzimidazoles Directly from Aromatic Diamines and Alcohols Catalyzed by Molecularly Defined Nonphosphine Manganese(I) Complex. *J. Org. Chem.* **2018**, *83*, 9553–9560.

3S. Shi X.; Guo, J.; Liu, J.; Ye, M.; Xu, Q. Unexpectedly Simple Synthesis of Benzazoles by tBuONa- Catalyzed Direct Aerobic Oxidative Cyclocondensation of o-Thio/ Hydroxy/Aminoanilines with Alcohols under Air. *Chem. Eur. J.* **2015**, *21*, 9988 – 9993.

4S. Dhanamjayulu, P.; Boga, R.; B., Mehta, A. Inhibition of aflatoxin B1 biosynthesis and down regulation of aflR and aflB genes in presence of benzimidazole derivatives without impairing the growth of *Aspergillus flavus*. *Toxicon* **2019**, *170*, 60–67.

5S. Moszczynski-Petkowski, R.; Majer, J.; Borkowska, M.; Bojarski, Ł.; Janowska, S.; Matłoka, M.; Stefaniak, F.; Smuga, D.; Bazydło, K.; Dubiel, K.; Wieczorek, M. Synthesis and characterization of novel classes of PDE10A inhibitors - 1H-1,3-benzodiazoles and imidazo[1,2-a]pyrimidines. *E. J. Med. Chem.* **2018**, *155*, 96-116.
